# Effect of azadirachtin pre-treatment on major pests, diseases and yield of seed yam

**DOI:** 10.5897/JEN2018.0222

**Published:** 2019-02-28

**Authors:** Mochiah Moses Brandford, Osei Kingsley, Aidoo Atta Snr, Danso Yaw, Lamptey Joseph Nii Lante, Ennin Stella Ama

**Affiliations:** CSIR-Crops Research Institute, P. O. Box 3785, Kumasi, Ghana

**Keywords:** Arthropod pests, biopesticides, *Dioscorea* spp, nematodes, synthetic pesticide

## Abstract

Pests and diseases infested material of seed yams have led to sub optimal yield levels. Neem was applied as substitute for chemical pesticides and as environmentally safe bio-pesticide to reduce the crops annual loss due to herbivore pests and their resulting diseases. Field trials were conducted during the 2016 and 2017 major planting seasons in four communities each of Ejura-Sekyedumase and Atebubu-Amantin districts of the Ashanti and Brong Ahafo regions respectively. Yam minisetts sizes were pre-treated with Mancozeb at a rate of 100 g and Lambda cyhalothrin at 40 ml in 10 L water as cocktail. Yam minisetts of 30 g each were planted on ridges at 100 cm between and 30 cm within rows on a 20 m × 20 m plot size. Neem leaf powder was applied on five rows per plot and another five served as check for assessment. Harvesting was done approximately 6 to 7 months after planting. Scale insects, mealybugs, beetles, termites and millipedes infestations. Galling due to root-knot nematodes, cracks, soft and wet rot were assessed on a scale of 1-5. Tuber yields were also assessed. Neem treated plots were observed to have significantly reduced arthropod pest populations and nematode galling as well as damage signs compared to the control plots. Yields of seed yam were higher on the neem treated plots than the control, probably due to the reduced damage on the treated plots. Seed yam yield increased for plots treated with *Azadirachta indica* leaves by 40 and 41% at Ejura-Sekyedumase district as well as 45 and 20% at Atebubu-Amantin district for 2016 and 2017 respectfully. Plots of seed yams treated with neem recorded reduced pests and damages in terms of parameters measured and subsequently translated into yield. It is paramount to seed yam producers to adopt neem leaf powder as pre-treatment for higher productivity.

## INTRODUCTION

Yam (*Dioscorea* spp.) is one of the most important root and tuber bearing crop in the world and an important source of energy. More importantly, yam tubers have organoleptic qualities which make them the preferred carbohydrate staple and can contribute up to 350 dietary calories per person each day (Asiedu et al., 2003). In Ghana, yams are used for local dishes. The two most cherished dishes are “fufu” (yam pounded into a thick paste after boiling and eaten with soup) and “ampesi” (boiled yam eaten with sauce). Besides nutritional significance, yam has pharmaceutical properties (Benghuzzi et al., 2003). It is a staple food for millions of people in tropical countries, notably in West Africa, the Caribbean’s and parts of southeast Nigeria (Hgaza et al., 2010). *Dioscorea* species with nutritive and antioxidant content (such as superoxide dismutase) not only enrich the diet of consumers but also make them ethnomedicinally important (Chandrasekara and Kumar, 2016; Liu et al., 2016). The socio-cultural significance of yam cannot be over emphasized. Yam is extensively produced in West Africa where they are steeped in cultural history and revered as a cultural symbol of fertility (Bridge et al., 2005). Yam serves as a special component of traditional gift, fine and bride price (IITA, 2004). It is also used in curing gastritis among Yoruba local groups of Cuba (Kadiri et al., 2014).

In Ghana, large-scale cultivation of the crop is in the Afram plains, the three northern, Brong Ahafo and Ashanti regions (Osei et al., 2011). Production of yam offers employment, food, cash and medicine (Aighewi et al., 2014) for people. There is ready market for the crop as there is a high demand on both the local and export markets (Armah, 2010). The importance of quality seed yams in the production of the root crop is important because seed yams are generally prone to pests and diseases infections (Mbiyu et al., 2012). Ghana loses about 40% of its yam production to diseases and pests (Aighewi et al., 2015). Yam farmers at a workshop in the Northern Region of Ghana in 2012 reported 10% loss by anthracnose diseases and 50% loss by other diseases such as nematodes (Peters et al., 1999). In West Africa, arthropod pests and plant-parasitic nematodes damage are major constraint to yam tuber quality reduction, yield losses in the field and storage. The major nematode pests include the yam nematode, *Scutellonema bradys*, and root-knot nematode, *Meloidogyne* spp. which are field and post-harvest pests (Agbaje et al., 2003; Adegbite et al., 2005). Application of synthetic pesticides is the most effective control strategy in yam seed production but these chemicals are expensive compared to botanicals as demonstrated in a study by Amoabeng et al. (2014). Synthetic pesticides can impact negatively on the environment, untargeted pests, microorganisms and human (Bell, 2000; Aktar et al., 2009). Organic soil amendments provide beneficial effects which include nematicidal properties and the potential to increase the yields of crops significantly (Rivera and Ballay, 2008). Farmers in Ghana customary have used plant like *Azadirachta indica* (Sapindales: Meliaceae) as traditional products for crop protection (Gerken et al., 2001). Oil extracted from *A. indica* (neem) seed has insecticidal and medicinal properties (Hassan et al., 2010).

Considering the negative effects usually posed on the environment and on non -target organisms, there is a public outcry for a paradigm shift from the use of synthetic pesticides to the use of better alternative methods such as plant extracts which are safe, cost effective, and feasible for crop protection (Amadioha, 2000). The use of healthy seeds yam in the system will go a long way to increase yam productivity in the sub region as it contributes to lowering of pests and diseases potential load in the environment. The objective of this study therefore, is to evaluate the effect of neem leaf powder application in managing arthropod pests and plant parasitic nematodes damage to seed yam.

## MATERIALS AND METHODS

### Study area

Field demonstration trials were conducted during the 2016 and 2017 major yam planting seasons in each of the four communities per Ejura Sekyedumase and Atebubu Amantin districts of the Ashanti and Brong Ahafo regions respectively (Table 1). These districts belong to the forest-savanna transitional zone with an average temperature that ranged from 21.5 to 30.7°C and relative humidity (RH) that ranged from 60 to 84% (MoFA, 2011). The annual rainfall is between 1200-1600 mm and the soil type is described as Orthic-Ferric Acrisols (well drained sandy clay loam soil) (Adu and Asiamah, 1992). These districts possess prominent yam farming communities where the crop is intensively cultivated. They have promising local markets and experience bimodal rainfall pattern (Owusu-Danquah et al., 2015).

**Table 1:** GPS Coordinates of the four communities within two districts of the study area

Region	District	Community	GPS Coordinate	Altitude (m)
Ashanti	Ejura-Sekyedumse	Bisiw	7° 19'42.916"N 1°17'38.916"W	202.31
		Nhyinase	7° 35'13.965"N 1°21'20.321"W	176.76
		Kramokrom	7° 19'32.171"N 1°16'15.846"W	195.55
		Mesuo	7° 32'15.351"N 1°18'13.087"W	241.82
Brong Ahafo	Atebubu- Amantin	Mem	7° 41'06.313"N 0° 58'25.522"W	164.80
		Abour	7° 35'54.045"N 1° 06'25.696"W	199.27
		Asanteboa	7° 38'36.536"N 1° 05'19.865"W	184.72
		Watro	7° 36'47.530"N 0° 57'57.757"W	175.92

### Experimental design, planting and treatment

Four prominent yam growing communities were randomly selected in each district with each community serving as a replication. Yam minisetts were pre-treated with 100 g of ethylene bisdithiocarbamate (fungicide) and Lambda cyhalothrin (insecticide) at 40 ml in 10 L of water (that is, 40/10000:v/v) mixed as cocktail. Mini sett sizes, 30 g each were planted on ridges at 100 cm between and 30 cm within rows on a 20 m × 20 m plot size. Neem leaf powder was applied to the soil on five rows per plot and another five served as check (control) for assessment. Air-dried neem leaves were milled using corn mill (Model no. 1A/2a, Crossword Agro Industries, India) and applied at 20 g per hill, which was placed in the planting hole and covered with a little soil before placing the yam minisett. The trial was weeded three times manually (one, three and six months) before harvesting. Harvesting was done approximately six to seven months after planting with the aid of hoe and cutlass.

### Data collection

Twenty seed yams were randomly selected and harvested from the neem treated and the control plots. Scale insects (*Aspidiella hartii*), mealybugs (*Planococcus citri*), beetles (*Heteroligus meles*), termites (*Amitermes* spp., *Macrotemes* spp. and *Microtermes* spp.)(Isoptera: Termitidae) and millipedes (*Plethocrossus* sp) (Diplopoda: Odontopygidae) infestations as well as nematode galling, cracks, soft and wet rot were assessed on the scale of 1-5; where 1 = free from attack, 2 = 1-25%, 3 = 26-50%, 4 = 51-75% and 5 = 76-100%. The tuber yield ton per hectare (t/ha) was also assessed and recorded.

### Statistical analysis

Data obtained on number of feeding holes created by scale insects, mealy bugs, beetles, termites and millipedes infestations on tubers and tuber weight expressed in yield were analyzed by one-way analysis of variance (ANOVA) with SAS, PROC GLM (SAS Institute Inc., 2005). Both arthropod damage and nematode galling index data were normalized using log10 (*x* +1) transformation. Percentage data was transformed using arcsine of *x* prior to analysis. Means were separated using Tukey´s test at 0.05 probability level.

## RESULTS

Major soil arthropods and their damages recorded in this study from both Ejura-Sekyedumse and Atebubu districts included scale insects *A. hartii*) Cockerel, the mealy bug *P. citri* (Risso) (Hemiptera: Pseudococcidae), yam tuber beetle (*H. meles* Billberger) holes, termites (*Amitermes* spp., *Macrotemes* spp. and *Microtermes* spp.) and millipedes (*Plethocrossus* sp) holes.

The results from Ejura-Sekyedumse district in 2016 indicated that, neem application had no significant effect (p > 0.05) on the scale insects and millipede holes. However, a significant difference (p<0.05) on the number of mealy bugs, termites and tuber beetle holes was observed when neem was applied (Table 2a). Considering nematodes and rot damages, in 2016, with the exception of galling there was no significant (p > 0.05) differences in the parameters measured such as crack, dry rot and wet rot (Table 2b).

**Table 2a:** Mean (± SE) of arthropod pest on seed yam at harvest from Ejura-Sekyedumse district, 2016.

Treatment	Scale insects	Mealy bugs	Termites	Beetle tuber holes	Millipede tuber holes
Neem	1.07±0.02	1.00±0.00^b^	1.29±0.04^b^	1.40±0.03^b^	1.09±0.01
No neem	1.10±0.02	1.10±0.02^a^	1.51±0.03^a^	1.52±0.06^a^	1.09±0.01
P>F	0.3743	0.0048	0.0001	0.0484	0.8606

Note: Means with the same letters within columns are not significantly different at 5% level of probability using Tukey´s test.

**Table 2b:** Mean (± SE) of Nematode and rot damages on seed yam at harvest from Ejura-Sekyedumse district, 2016

Treatment	Crack	Galling	Dry rot	Wet rot
Neem	2.24±0.03	2.10±0.02^b^	2.06±0.02	2.05±0.02
No neem	2.26±0.02	2.21±0.02^a^	2.06±0.01	2.07±0.01
P>F	0.6158	0.0020	0.6954	0.2443

Note: Means with the same letters within columns are not significantly different at 5% level of probability using Tukey´s test.

Results from Atebubu- Amantin district in 2016 indicated that neem application had no significant (*p* > 0.05) effect on the scale insects, termite and millipede holes. However, significant (p < 0.05) difference on the number of mealy bugs and tuber beetle holes was observed when neem was applied (Table 3a). At Ejura-Sekyedumse in 2016, similar trend was observed for nematode and rot damages. Galling was the only parameter that showed no significant (P > 0.05) difference. Crack, dry rot and wet rot as parameters recorded significant (p < 0.05) differences on neem treated plots (Table 3b).

**Table 3a:** Mean (± SE) of arthropod pest on seed yam at harvest from Atebubu- Amantin district, 2016.

Treatment	Scale insects	Mealy bugs	Termites	Beetle tuber holes	Millipede tuber holes
Neem	1.01±0.00	1.00±0.00^b^	1.05±0.01	1.09±0.01^b^	1.00±0.00
No neem	1.02±0.00	1.01±0.00^a^	1.07±0.01	1.16±0.01^a^	1.01±0.00
P>F	0.5095	0.0046	0.2817	0.0001	0.1730

Note: Means with the same letters within columns are not significantly different at 5% level of probability using Tukey´s test.

**Table 3b:** Mean (± SE) of nematode and rot damages on seed yam at harvest from Atebubu- Amantin district, 2016.

Treatment	Crack	Galling	Dry rot	Wet rot
Neem	2.06±0.01^b^	2.08±0.01	2.02±0.01^b^	2.00±0.00^b^
No neem	2.11±0.01^a^	2.08±0.01	2.05±0.01^a^	2.02±0.00^a^
P>F	0.0001	0.4979	0.0481	0.0013

Note: Means with the same letters within columns are not significantly different at 5% level of probability using Tukey´s test.

The results from Ejura-Sekyedumse district in 2017 indicated that, neem application had no significant (*P* > 0.05) effect on the scale insects and mealy bugs.

However, there was significant (p < 0.05) difference for three parameters; termite tuber beetle holes and millipede holes when neem was applied (Table 4a). For nematode and rot damages, in 2017, cracks and wet rot were the only two parameters that showed no significant (p > 0.05) differences. Galling and dry rot, however, recorded significant (p > 0.05) differences on neem treated plots Table 4b.

**Table 4a:** Mean (± SE) of arthropod pest on seed yam at harvest from Ejura-Sekyedumse district, 2017.

Treatment	Scale insects	Mealy bugs	Termites	Beetle tuber holes	Millipede tuber holes
Neem	1.04±0.01	1.00±0.00	1.29±0.04^b^	1.34±0.03^b^	1.04±0.01^b^
No neem	1.07±0.02	1.03±0.01	1.48±0.03^a^	1.52±0.06^a^	1.09±0.03^a^
P>F	0.2691	0.1471	0.0008	0.0022	0.0311

Note: Means with the same letters within columns are not significantly different at 5% level of probability using Tukey´s test.

**Table 4b:** Mean (± SE) of nematode and rot damages on seed yam at harvest from Ejura-Sekyedumse district, 2017.

Treatment	Crack	Galling	Dry rot	Wet rot
Neem	2.24±0.03	2.10±0.02^b^	2.02±0.01^b^	2.03±0.01
No neem	2.21±0.02	2.17±0.02^a^	2.06±0.02^a^	2.04±0.02
P>F	0.4075	0.0281	0.0140	0.6092

Note: Means with the same letters within columns are not significantly different at 5% level of probability using Tukey´s test.

The results from Atebubu- Amantin district in 2017 indicated that, neem application had no significant (*p* > 0.05) effect on the scale insects and tuber beetle holes. Mealy bugs, termite and millipede holes, however, showed significant (*P* < 0.05) difference as parameters when neem was applied (Table 5a). For nematode and rot damages, in 2017, cracks and galling as parameters showed significant (p < 0.05) difference while dry rot and wet rot as parameters recorded no significant (p > 0.05) differences on neem treated plots (Table 5b).

**Table 5a:** Mean (± SE) of arthropod pest on seed yam at harvest from Atebubu- Amantin district, 2017.

Treatment	Scale insects	Mealy bugs	Termites	Beetle tuber holes	Millipede tuber holes
Neem	1.00±0.00	1.15±0.04^b^	1.08±0.03^b^	1.12±0.05	1.04±0.02^b^
No neem	1.00±0.00	1.22±0.11^a^	1.17±0.03^a^	1.12±0.02	1.13±0.02^a^
P>F	-	0.0013	0.0003	0.9169	0.0015

Note: Means with the same letters within columns are not significantly different at 5% level of probability using Tukey´s test

**Table 5b:** Mean (± SE) of nematode and rot damages on seed yam at harvest from Atebubu- Amantin district, 2017.

Treatment	Crack	Galling	Dry rot	Wet rot
Neem	2.10±0.04^b^	2.05±0.02^b^	2.01±0.01	2.01±0.01
No neem	2.17±0.02^a^	2.11±0.02^a^	2.03±0.01	2.03±0.01
P>F	0.0081	0.0061	0.4189	0.2511

Note: Means with the same letters within columns are not significantly different at 5% level of probability using Tukey´s test.

For the two growing and harvesting years of 2016 and 2017, there were significant differences (P < 0.05) between the neem treated plots and the control. Yields of seed yam/tubers were higher on the neem treated plots than the control (Table 6). Generally, yield from Ejura-Sekyedumse out performed that of Atebubu-Amantin The highest yield (16.04 and 15.38 t/ha) per plot (weight) was recorded by the neem treated plots from Ejura-Sekyedumse whilst control (untreated) plots recorded the least yield (7.67 and 8.06 t/ha) from Atebubu-Amantin for the two growing seasons (Table 6).

**Table 6:** Mean (± SE) of yield from on seed yam/tubers at harvest from Ejura-Sekyedumse and Atebubu-Amantin district, 2016 and 2017.

Treatment	Mean yield (t/ha) for 2016		Mean yield (t/ha) for 2017	
	Ejura-Sekyedumse	Atebubu-Amantin	Ejura-Sekyedumse	Atebubu-Amantin
Neem	16.04 ± 1.02^a^	13.99 ± 1.54^a^	15.38 ± 1.03^a^	9.92±1.74^a^
No neem	9.67 ± 0.63^b^	7.67 ± 0.86^b^	9.00 ± 0.7^4b^	8.06±1.71^b^
P>F	0.0001	0.0001	0.0001	0.0298

Note: Means with the same letters within columns are not significantly different at 5% level of probability using Tukey´s test.

## DISCUSSION

Results from this study, generally indicated that neem treated plots were observed to have significantly reduced arthropod pest populations and nematode infestations. These were observed on neem treated plots compared to the control. In the field, pests and pathogens were found to have significantly contributed in seed yam losses. The arthropod pests namely scale insects, mealy bugs, yam tuber beetles, millipedes and nematodes (*S. bradys*, *Pratylenchus* spp. and *Meloidogyne* spp.) were responsible for tuber damages. They probably might have served as primary invaders which facilitated fungal (e.g. *Botryodiplodia* spp., *Aspergillus* spp., *Fusarium* spp.) pathogen tuber infection in the field. The resulting damages that occurred in the field might have led to reducing the quality, quantity of food and planting material if neem were not applied. Similar findings have been earlier reported by Amusa et al. (2003) and Ogaraku and Usman (2008) where most of the losses originated from pre-harvest invasion or infection and/or damage (Morse et al., 2000). Some authors have contended that application of neem (Azadiractin) decreased pest populations when used to control *Meloidogyne* spp. (root-knot nematodes), *Rhizoctonia* root-rot fungus and rice stunt virus (Anonymous, 1992; Anjorin et al., 2004).

Population of the arthropod pests, tuber damage and rots in the present study was significantly reduced by the application of the neem. Presumably, proportional number of tubers were observed to have more numbers of damaged holes caused by pests on untreated plots. According to Pwakem (2015), damage holes that are left on the tubers by yam beetle, *H. meles* are serious on yam from marshy areas particularly in the forest zones, up to the savanna region in yam producing districts of Ghana. In the forest and Guinea savanna zones of Nigeria, the yam beetle has also been found as major constraint to yam production (Tobih et al., 2007a; Okoroafor et al., 2009)

The beetle feeds and creates spherical and semispherical lesions of varying sizes on tubers in the range of 1 to 20 holes in a tuber (Tobih et al., 2007b; Okoroafor et al., 2009). These lesions predispose tubers to fungal and bacterial attacks both before and after harvest under suitable environmental conditions (Morse et al., 2000). The feeding of *H. meles* cause about 22-77% tuber loss, drastic reduction of yield and market value, and damage in extreme cases resulting from adult feeding on yam can cause plant death (Taylor, 1964). It is important to control this beetle on yams since yams have significant health and economic value (Coursey, 1967).

Results from the two yam producing districts in 2016 and 2017 indicated that all the parameters rated on a score from 1 to 5 on both neem treated and untreated plots recorded moderately low value since none was above the rating score of scale 3. The application of neem to manage nematodes has been reported in several studies (Nazir et al., 2006; Osei et al., 2013; Kankam and Adomako, 2014). Neem application contributed to yield improvement and reduced the effect of arthropod pests and nematodes stress. This indicates the relevance of employing another beneficial approach in managing biotic stress to improve productivity of farmer saved seeds. The system of saving seed yams and using them as planting materials for the following season can lead to endemic pathogens and persist in the tubers over generations when they are left untreated at planting, harvesting and beyond (Yam and Arditti, 2009). The adoption of neem leaf powder application has the potential to enhance the yam export trade and generally re-vitalize the industry in yam producing countries.

Yam has currently gained attention on the export market and there are premium prices for food commodities that do not have pesticide contamination which health-conscious consumers eagerly patronise (Njoroge and Manu, 1999). The result of this study on neem application has the advantage of increasing yield at low cost which is crucial to resource-poor smallholder farmers. Furthermore, yam growers can confidently rely on neem to manage pests and diseases in the wake of increasing awareness of health hazards of insecticide-contaminated food commodities. Furthermore, consumers are gradually changing their perception on how food commodities are produced even in the developing countries. For example, consumers in Ghana and Benin have expressed desire to pay more than 50% premium prices for vegetables that will be certified as free from pesticide contamination (Coulibaly et al., 2007). In addition, organic food producers and yam exporters could use neem as an added advantage to gain access to the USA and the EU markets where strict compliance to pesticide levels in food commodities is a requirement (Njoroge and Manu, 1999).

Yields of seed yam/tubers were higher on the neem treated plots than the control, probably due to the reduced damage on the treated plots. Farmers from Ghana and sub region can take advantage of botanicals usage and increase marketable yields of yam tubers by applying neem to the soil as pre-treatment. Salako et al. (2008) observed that neem applications before planting of the yam setts improved germination and increased yield from 12 to 28 Mt/ha. In the same study, score on scale insect is observed to have been reduced significantly. The efficacy of *A. indica* leaf powder in this study might be attributed to the presence of bioactive components such as Vilasinin from green leaves, 6-Acetylnimbandiol, 6-Acetyl-niminene reported to be present in the leaf and can be utilized as pesticide (Gopinathan, 2007).

 Amoabeng et al. (2014) demonstrated that Siam weed and tobacco extracts gave significantly higher undamaged cabbage head yields and commensurately more favourable economic benefit than emamectin benzoate (Attack®). This paper support the assertion that smallholder farmers especially those in the developing countries like Ghana, who have free access to such plant materials and have the labour availability stand to gain immensely. The use of synthetic insecticides has been linked with causing hazards to humans, animals and the environment. Botanicals are generally regarded as safer to users, consumers, animals and the environment due to their non-persistent nature (Buss and Park-Brown, 2002). In contrast, synthetic insecticides are often inaccessible to resource-limited farmers or are hazardous to use due to poor access to safety equipment and adequate training in safe use.

Availability of good and quality planting materials is essential to sustain high production of yams in Ghana. To avert or reduce the negative impacts of chemical residual effect, safer alternative approaches to managing pests of yams like application of neem must be considered by growers, especially those who do not have the expertise and equipment for safe handling and use of synthetic insecticides (Ntow et al., 2006; Coulibaly et al., 2007). In developing countries such as Ghana, food commodities often contain pesticide residues, often above the maximum residue limit (Darko and Akoto, 2008; Armah, 2011). The reliance on botanical products for pre- and post-harvest treatment of food commodities has the potential to preserve the health of consumers and the environment alike.

## CONCLUSION

Seed yams materials treated with neem significantly reduced arthropod pests and nematodes infestations and subsequently translated into yield increase. As additional information, seed yam producers are advised to avoid late harvesting which predisposes the tubers to attack by soil arthropods and diseases that will affect the storability and seed value of the tubers. Finally, application of neem is recommended as substitute for chemical pesticides as environmentally safe bio-pesticide to reduce the crops annual loss due to herbivore pests (and their resulting diseases), as well as increasing the food security in Africa.

We also observed that late harvesting predisposes tubers to bruises and attack so seed yam producers are advised to be early to attain maturity.
